# Primary hepatopancreatobiliary lymphoma: Pathogenesis, diagnosis, and management

**DOI:** 10.3389/fonc.2022.951062

**Published:** 2022-08-30

**Authors:** Qianwen Wang, Kangze Wu, Xuzhao Zhang, Yang Liu, Zhouyi Sun, Shumei Wei, Bo Zhang

**Affiliations:** ^1^ Department of Surgery, Fourth Affiliated Hospital, School of Medicine, Zhejiang University, Yiwu, China; ^2^ Department of Surgery, Second Affiliated Hospital, School of Medicine, Zhejiang University, Hangzhou, China; ^3^ Department of Hematology, Second Affiliated Hospital, School of Medicine, Zhejiang University, Hangzhou, China; ^4^ Department of Pathology, Second Affiliated Hospital, School of Medicine, Zhejiang University, Hangzhou, China

**Keywords:** primary hepatic lymphoma, primary biliary lymphoma, primary pancreatic lymphoma, diagnosis, treatment

## Abstract

Primary hepatopancreatobiliary lymphoma (PHPBL) is extremely rare, which is defined as a lympho-proliferative disease confined to the hepatobiliary system and pancreas without any involvement of lymph nodes, bone marrow, or other organs. The clinical and imaging manifestations of PHPBL are variable and non-special, which are akin to those of tumors of the hepatobiliary and pancreatic systems. The overall prognosis and management of PHPBL differ from those of other tumors in the hepatobiliary system and pancreas. Proper diagnosis and prompt treatment are essential for improving clinical outcomes. Due to its rarity, the optimal treatment has not been issued. However, combination chemotherapy is considered as a standard treatment for them. This review provides an overview of the pathogenesis, diagnosis, pathology, and management of PHPBL and offers clinicians the diagnosis and management schedule for PHPBL.

## Introduction

Lymphoma accounts for about 4.8% of newly diagnosed cases of cancer in the United States, 90% of which is non-Hodgkin lymphoma (NHL), and about 3.6% of all cancer deaths (1). Lymphoma is traditionally classified as Hodgkin lymphoma (HL) and NHL. They can both locate in sites except for the lymphatic system, which is commonly found in NHL (2). The most common site of extranodal involvement is the gastrointestinal tract, particularly stomach and small bowel (3). Involvement of hepatobiliary and pancreatic system is rare and is divided into primary and secondary lymphoma (4). Primary hepatopancreatobiliary lymphoma (PHPBL) is a rare entity, which has a lower incidence than secondary hepatopancreatobiliary lymphoma (SHPBL), commonly presented in widespread lymphoma.

The commonly accepted diagnosis criteria for PHPBL, as defined by previous studies, include a mass in the hepatobiliary and pancreatic systems, no enlargement of superficial and mediastinal lymph nodes, no involvement of other organs, and normal leukocyte count in the peripheral blood smear (5, 6).

Primary hepatic lymphoma (PHL), primary biliary lymphoma (PBL), and primary pancreatic lymphoma (PPL) are rare and account for 0.4%, 0.4%, and 1% of extranodal non-Hodgkin’s lymphomas, respectively (7–9). PHL and PPL both affect middle-aged people, with a marked male preponderance, whereas PBL often affect elderly individuals, with a sight female preponderance (10–12). Due to their non-specific clinical manifestations and radiological features, PHPBL is always confused with other diseases of the hepatobiliary system and pancreas. The discrepancy in their treatment and prognosis makes it important to achieve a proper diagnosis.

In this article, we will discuss the pathogenesis, epidemiology, clinical presentation, imaging feature, pathological finding, and treatment of PHPBL (**Tables 1**–**3**). We aim to raise clinicians’ awareness of the possibility of PHPBL, when they meet a patient with a mass in the hepatobiliary system and pancreas, and offer them the diagnosis and management schedule for PHPBL.

**Table 1:** Summary of the main features of primary hepatic lymphoma.

Histotype	Clinical manifestations	Imaging findings	Pathology	Immunophenotype	Behavior	Treatment
DLBCL	Abdominal pain; B symptom; nausea, vomiting; jaundice	A solid mass/multiple masses	Diffuse infiltration of large lymphocytes with prominent nucleoli and increased or atypical mitotic figures	CD20+, CD79a+, CD19+, CD3- GC group: CD10+ or/and BCL-6+, MUM-1- Non-GC group: MUM-1+, BCL-6-, CD10-	Aggressive	Surgery+ chemotherapy (CHOP/R-CHOP) +/- radiation; chemotherapy; surgery
BL			Diffuse infiltration of medium-sized lymphocytes, with distinct nucleoli, scant cytoplasm, and increased mitotic figures; starry-sky appearance	Monotypic sIgM+, CD20+, CD10+, CD43+, BCL-6+; CD3-, CD5-, CD23-, BCL-2-		
MALT		A solid mass/multiple masses in portal fields of the liver	Diffuse infiltration of small-sized lymphocytes, with mildly irregular nucleoli, dense chromatin and scant cytoplasm, without germinal center differentiation; lymphoepithelial lesions in bile duct	CD20+, CD79α+, Bcl-2+, CD21+ CD3-, CD10-, CD5-, CD23-, CD43-, and cyclinD1-	Mostly indolent	
FL		A solid mass/multiple masses	Distinct follicles, formed by small-to-medium-sized centrocytes; follicular meshworks	CD20+, CD10+, BCL-2+, BCL-6+, CD23+, CD21+		

*DLBCL, diffuse large B-cell lymphoma; BL, Burkitt lymphoma; MALT, mucosa-associated lymphoid tissue lymphoma; FL, follicular lymphoma.

**Table 2:** Summary of the main features of primary biliary lymphoma.

Histotype	Clinical manifestations	Imaging findings	Pathology	Immunophenotype	Behavior	Treatment
DLBCL	Obstructive jaundice; abdominal pain; B symptom; nausea, vomiting	A solid mass/multiple masses	Diffuse infiltration of large cell with prominent nucleoli, numerous mitoses	CD20+, CD79a+, CD19+, CD3-, CD45- GC group: CD10+ or/and BCL-6+, MUM-1- Non-GC group: MUM-1+, BCL-6-, CD10-	Aggressive	Surgery+ chemotherapy (CHOP/R-CHOP) +/- radiation; chemotherapy; surgery
MALT		An irregular thickening in the gallbladder or bile duct wall	Diffuse infiltration of small cell with intact mucosal layer and occasional lymphoid follicles; lymphoepithelial lesions	CD20+, CD79a+, CD19+, BCL-6-, CD10-, cyclin D1-	Mostly indolent	
FL			Infiltration of small or medium cleaved lymphocytes, forming follicles; follicular meshworks	CD20+, CD10+, BCL-2+, CD23+, CD3-, CD5-		

*DLBCL, diffuse large B-cell lymphoma; MALT, mucosa-associated lymphoid tissue lymphoma; FL, follicular lymphoma.

**Table 3:** Summary of the main features of primary pancreatic lymphoma.

Histotype	Clinical manifestations	Imaging findings	Pathology	Immunophenotype	Behavior	Treatment
DLBCL	Abdominal pain; jaundice; B symptoms; acute pancreatitis; duodenal obstruction; nausea, vomiting;	A solid mass/multiple masses/diffuse enlarged pancreas without marked dilation of pancreatic duct and vascular infiltration	Diffuse infiltration of large atypical B cells with round nuclei, dispersed chromatin, some marked nucleoli, and scant cytoplasm	CD20+, CD79a+, CD19+, CD5-, cyclin D1-, CD30-, CD56- GC group: CD10+ or/and BCL-6+, MUM-1- Activated GC group: CD10+ or/and BCL-6+, MUM-1+ Activated non-GC group: CD10-, BCL-6-, MUM-1+	Aggressive	Surgery+ chemotherapy (CHOP/R-CHOP) +/- radiation; chemotherapy; surgery
FL			Infiltration of the small or medium-sized cleaved cells forming uniform and dense follicles	CD20+, CD10+, CD19+, BCL-2+, BCL-6+; CD3-, CD5-	Mostly indolent	
MALT			Infiltration of morphologically heterogeneous small B cells	CD20+, CD79α+, Bcl-2+, CD21+ BCL-6-, CD3-, CD10-, CD5-, and cyclinD1-		
BL			Diffuse infiltration of medium-sized lymphocytes, with round-to-irregular nucleoli, scant cytoplasm; starry-sky appearance	Monotypic sIgM+, CD20+, CD10+, and Bcl-6+; CD3-, CD5-, TdT-, Bcl-2-	Aggressive	

*DLBCL, diffuse large B-cell lymphoma; BL, Burkitt lymphoma; MALT, mucosa-associated lymphoid tissue lymphoma; FL, follicular lymphoma.

## Primary hepatic lymphoma

### Risk factors and pathogenesis

The exact risk factors are not clear and several studies have suggested that chronic liver diseases play an etiological role in the development of PHL, including Epstein–Barr virus (EBV), hepatitis B or C virus infection, liver cirrhosis, and primary biliary cirrhosis (13–17). Like gastric mucosa-associated lymphoid tissue (MALT) lymphoma, primary MALT lymphoma of liver may also be associated with *H. pylori* infection (18). In addition, PHL could present in patients with autoimmune diseases (19, 20), those with acquired immune deficiency syndrome (AIDS), and immunosuppressive drug-treated transplant recipients (21–23).

In all conditions, chronic inflammation of the liver represents the common steps in the pathogenesis of PHL, which induce lymphocyte migration to liver, mediated by some adhesion molecules, and cause B lymphocyte chronic proliferation, eventually leading to hepatic lymphoma (24, 25). Lack of T-cell surveillance is also cited as an inciting factor.

Among all these conditions, hepatitis C virus (HCV) infection is strongly associated with PHL (26), which causes malignant transformation by indirect methods. HCV, a lymphotropic virus, brings about chronic stimulation of B-cell and polyclonal proliferation, eventually leading to hepatic lymphoma. It may also induce a t (14, 18) translocation resulting in overexpression of BCL-2, an anti-apoptotic factor, and rearrangement of monoclonal IgH. Furthermore, through the viral core proteins, it can downregulate the transcription of tumor suppressor genes like p21, p53, and Ras (24).

**Figure 1:**
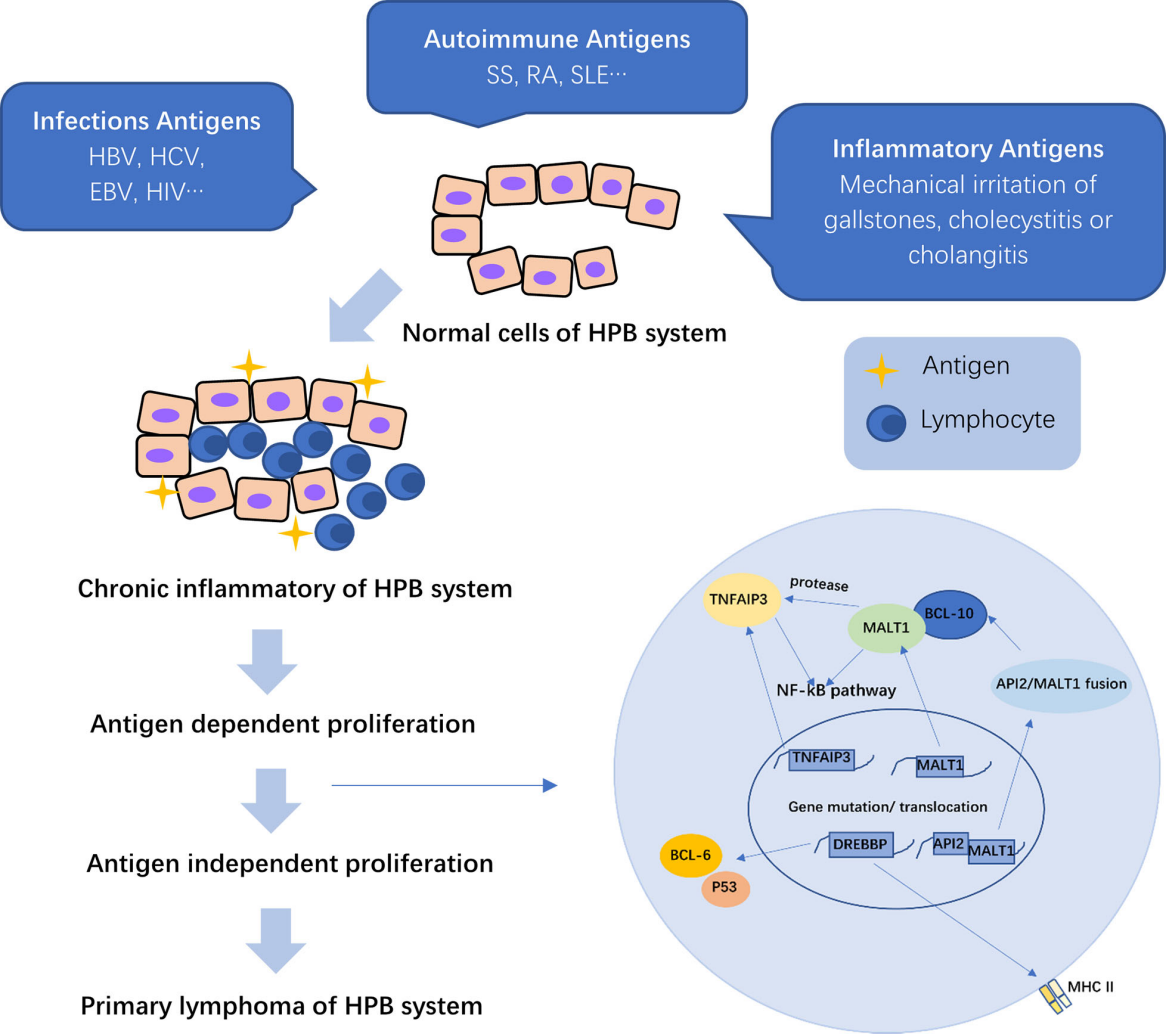
The pathogenesis of primary hepatopancreatobiliary lymphoma (PHPBL). As a result of the prolonged antigen exposure, chronic inflammation of the HBP (hepatopancreatobiliary) system induces lymphocyte migration to the site of inflammation and antigen-dependent proliferation. Prolonged inflammatory stimulation can cause irreversible chromosomal translocations and induce lymphocytes to antigen-independent proliferation, thus leading to primary lymphoma of HBP. MALT1, mucosa-associated lymphoid tissue 1; TNFAIP3, tumor necrosis factor alpha-inducible protein 3; CREBBP, cyclic adenosine monophosphate (cAMP) response element binding protein; API2, apoptosis inhibitor 2; MHC II, major histocompatibility complex II; NF-kB, nuclear factor-kappa B.

In primary hepatic MALT lymphoma, the most frequent translocation is t (14, 18) (q32; q21), which brings the mucosa-associated lymphoid tissue 1 (MALT1) gene to the downstream of the IgG enhancer and makes MALT1 overexpressed, thus resulting in activation of the NF-κB pathway. Meanwhile, activated MALT1 has protease activities, causing hydrolyzation of tumor necrosis factor alpha inducible protein 3 (TNFAIP3), which act as a NF-κB negative regulator, further promoting NF-κB activation. Overexpression of MALT1 and BCL-10 can upregulate the BAFF expression, thus enhancing the activation of the non-canonical NF-κB pathway (**Figure 1**) (27).

TNFAIP3 plays a key role in the regulation of several inflammation signaling pathways, which can negatively regulate the NF-κB pathway by inhibiting signals from various surface receptors to activate the signaling pathway. Therefore, the inactivating mutation or deletion of TNFAIP3 gene can downregulate its expression and reduce the repression of NF-kB activation (**Figure 1**) (18, 27). Such genetic abnormalities play an essential role in lymphomagenesis.

Cyclic adenosine monophosphate response element binding protein (CREBBP) is a transcriptional coactivator, which is involved in several signaling pathways. Due to the lack of acetylation, the mutant CREBBP protein can activate BCL-6 and decrease the tumor suppressor activity of p53, thus protecting atypical lymphocytes from apoptosis. Deletion of CREBBP gene can downregulate the expression of MHC-II gene, leading to immune escape of malignant cells for proliferation and invasion (**Figure 1**) (18, 28).

### Epidemiology and clinical features

PHL is extremely rare, which makes up only 0.4% of extranodal NHL, and 0.016% of all NHL (7). PHL affects individuals of varying age, but it is essentially a disease of middle-aged people (median age: 50 years old). Men are affected approximately twice as women (10).

Although the clinical features of PHL are wide-ranging, patients with PHL usually present with a complaint of upper right abdominal pain (29). The typical B symptoms of fever and weight loss can be found in one-third of all cases (24). Other symptoms include fatigue, anorexia, nausea, jaundice, and vomiting (30–33). On the physical examination of abdomen, tenderness in the upper right quadrant and hepatomegaly are the common presenting features. Splenomegaly occasionally can be observed in a few cases, as a consequence of hepatic dysfunction and portal hypertension (24, 31, 34).

Typical serum findings include variably elevated lactic dehydrogenase (LDH), bilirubin levels, as well as elevated liver enzyme levels (35, 36). The level of LDH, as a prognostic marker, increases in 30%–80% of all cases (24). The significantly increased LDH and normal tumor markers are useful clues in the diagnosis of PHL (37). Full blood counts are usually within the normal range unless the bone marrow or spleen is involved (38). Other occasional laboratory findings include monoclonal paraproteinemia and hypercalcemia, which is possibly the result of the secretion of calcitriol by lymphoma cells (39, 40).

### Imaging

The imaging features of PHL are wide-ranging, and are commonly evaluated by abdominal ultrasound (US), CT, and MRI. At imaging, PHL can appear as a solitary mass, multiple lesions, or diffuse infiltration (41), mimicking other liver diseases, such as hepatocellular carcinoma, cholangiocarcinoma, metastatic disease of liver, and hepatitis.

On US, PHL mostly appears as homogeneous hypoechoic lesions confined in the liver (30, 42). Anechoic lesion can occasionally be seen, which may be confused with cyst (43). If there is a mass in the porta hepatis, dilation of the intra- and extra-hepatic bile ducts can also be found (44). The manifestations of contrast-enhanced US show mild heterogeneous enhancement in the arterial phase and washout in the portal and late phases (45).

**Figure 2:**
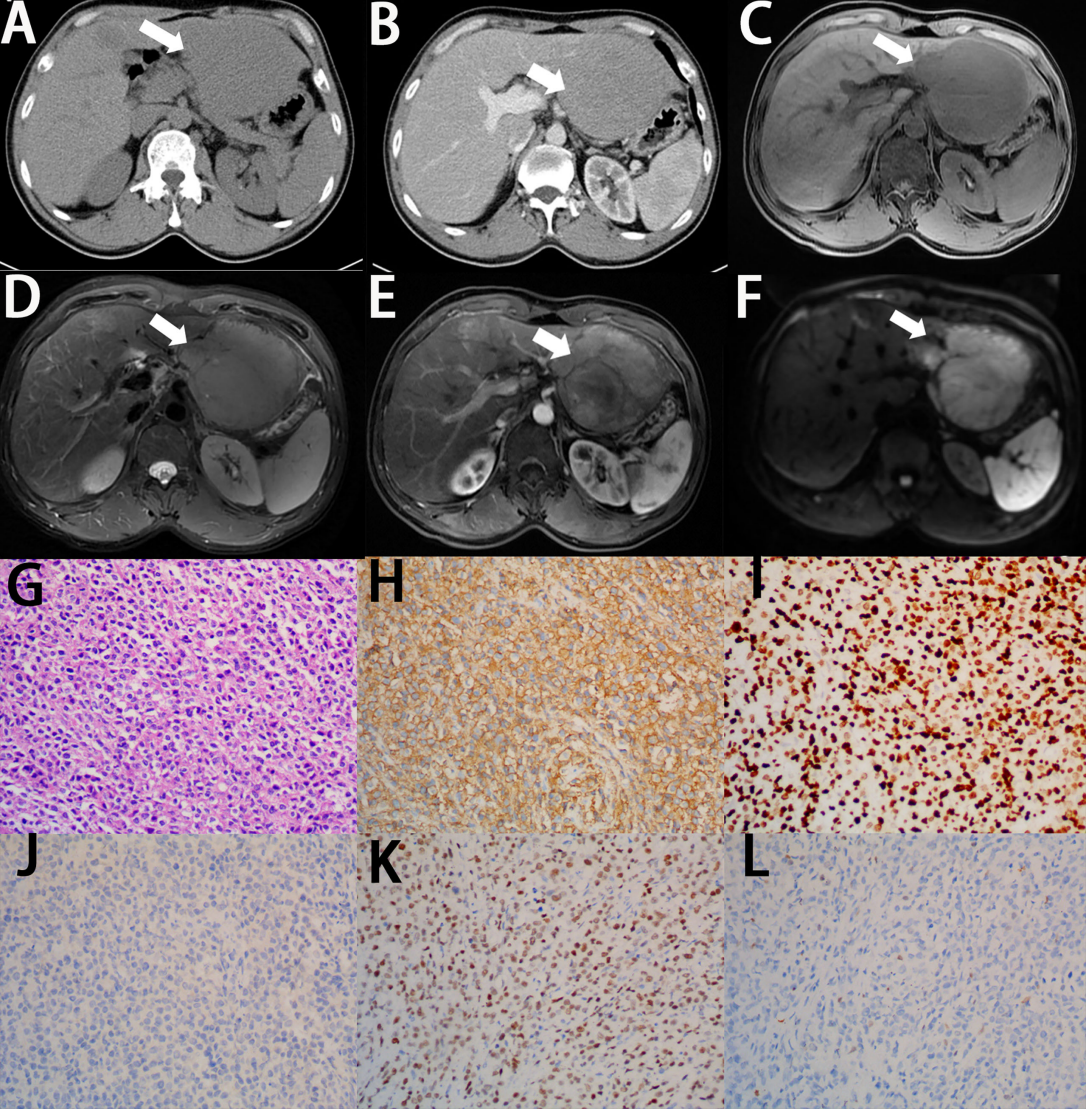
Imaging finding and histology of primary hepatic diffuse large B-cell lymphoma. **(A)** On unenhanced CT, the lesion shows hypoattenuation mass (white arrow). **(B)** On contrast-enhanced CT, the lesion shows a poor enhancement, lower than the normal parenchyma (white arrow). **(C)** On T1-weighted images, the lesion shows lower signal intensity (white arrow). **(D)** On T2-weighted images, the lesion shows higher signal intensity (white arrow). **(E)** On enhanced MRI, the lesion shows significant enhancement (white arrow). **(F)** On DWI, the lesion shows significant signal restriction (white arrow). **(G)** A diffuse infiltrate of large lymphocytes replaces the liver parenchyma (H&E stain, 20×). **(H)** Lymphocytes are positive for CD20 (CD20 immunostain, 20×). **(I)** The Ki-67 proliferative index is high in the lymphocytes (Ki-67 immunostain, 20×). **(J)** Lymphocytes are negative for CD10 (CD10 immunostain, 20×). **(K)** Lymphocytes are positive for BCL6 (BCL-6 immunostain, 20×). **(L)** Lymphocytes are negative for MUM-1 (MUM-1 immunostain, 20×).

**Figure 3:**
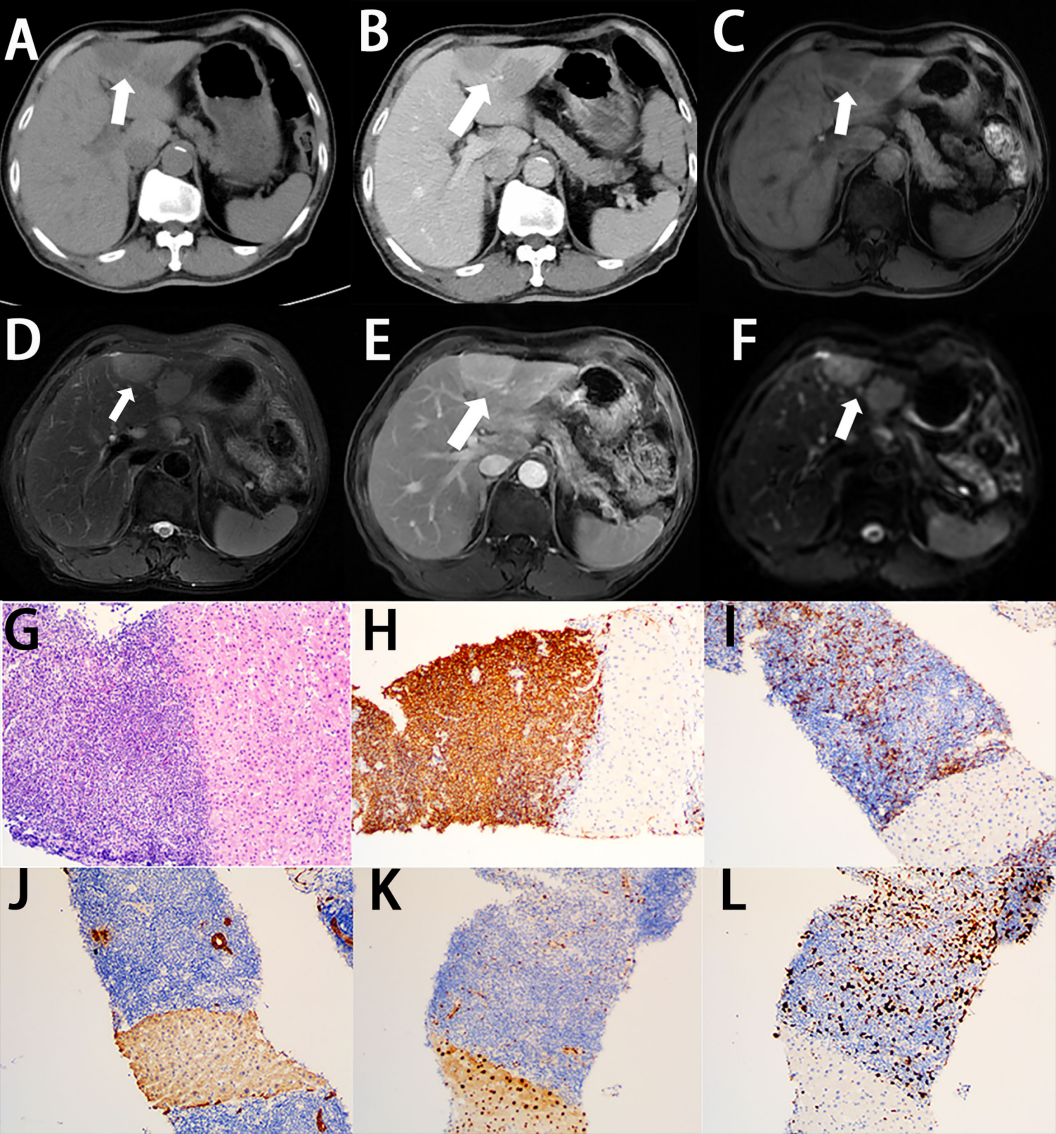
Imaging finding and histology of primary hepatic MALT lymphoma. **(A)** On unenhanced CT, the multiple lesions show hypoattenuation lesions (white arrow). **(B)** On contrast-enhanced CT, the multiple lesions show a poor enhancement, lower than the normal parenchyma (white arrow). **(C)** On T1-weighted images, the multiple lesions show lower signal intensity (white arrow). **(D)** On T2-weighted images, the multiple lesions show higher signal intensity (white arrow). **(E)** On enhanced MRI, the multiple lesions show significant enhancement (white arrow). **(F)** On DWI, the lesions show significant signal restriction. **(G)** A diffuse infiltrate of small-sized lymphocytes replaces the liver parenchyma (H&E stain, 10×). **(H)** Lymphocytes are positive for CD20 (CD20 immunostain, 10×). **(I)** A minor population of reactive T cells are positive for CD3 (CD3 immunostain, 10×). **(J)** Lymphocytes are negative for CKp (CKp immunostain, 10×). **(K)** Lymphocytes are negative for Cyclin D1 (Cyclin D1 immunostain, 10×). **(L)** The Ki-67 proliferative index is low in the lymphocytes (Ki-67 immunostain, 10×).

On CT, PHL always presents as hypoattenuating lesions, with or without distinct margins (**Figures 2A**, **3A**) (46, 47), the center of which may have a lower intensity, suggesting necrosis (2). However, it also presents as homogeneous or heterogeneous hepatomegaly without definite hepatic masses (48). Unlike hepatic carcinoma, PHL shows that hepatic vessels passed through the lesions without evidence of compression, or infiltration (48). On contrast-enhanced CT (CECT), the hepatic lesions show mild enhancement in the arterial phase (**Figures 2B**, **3B**) and progressive enhancement in the venous phase, which is vital to differentiate from hepatocellular carcinoma. Rim enhancement can also be noted (46, 47, 49).

On MRI, the lesions of PHL tends to show hypointense signal on the T1-weighted images (T1WI) and hyperintense signal on the T2-weighted images (T2WI) (**Figures 2C, D**, **3C,D**) (50, 51). Some PHLs show a heterogeneous signal because of necrosis or fibrosis within the mass (52). Nearly half of PHLs show enhancement after the intravenous administration of the contrast agent, which is lower than that of normal liver parenchyma (**Figures 2E**, **3E**), whereas 40% of PHLs are hypointense. PHL patients mainly present as a significant signal restriction in diffusion-weighted imaging (DWI), with a lower ADC value (median ADC value: 0.83 × 10^-3^ mm^2^/s) than other malignant hepatic diseases (**Figures 2F**, **3F**) (53). Like CT features of PHL, we can also observe no distortion of the blood vessels and bile ducts passing through hepatic lesions on MRI, which is called “insinuative growth” (54).

PET/CT has been an excellent modality for the diagnosis, staging, and follow-up of tumor, which can evaluate the involvement of other sites in patients with lymphoma and differentiate primary liver lesions from metastatic diseases (55, 56). The hypermetabolic lesions with a maximum Standardized Uptake Value (SUVmax, mainly ranging from 4.5 to 33.5) might be observed, with tumor confined in the liver without abnormal uptake in any other tissues or organs (57).

### Pathology and immunohistochemistry

The clinical and radiological findings of PHL are non-special, and a definite diagnosis can only be made after pathological analysis. Liver tissue can be obtained by fine needle biopsy (FNB), image-guided percutaneous biopsy, laparoscopic biopsy, or open biopsy (2, 58).

As the most common subtype of PHL, diffuse large B-cell lymphoma (DLBCL) accounts for up to 80% of all PHL cases (10), followed by MALT lymphoma, follicular lymphoma, Burkitt lymphoma, and T-cell lymphoma (24, 59–62).

Microscopically, on low-power magnification, the lymphoid cells infiltrate the hepatic parenchyma and form solitary, multiple nodules or diffuse infiltration (63). On high-power magnification, the histological features vary, depending on the subtypes of PHL. In primary hepatic DLBCL, the lesions are formed by diffuse large-sized lymphoid cells with prominent nucleoli and increased or atypical mitotic figures (**Figure 2G**) (64–66). In primary hepatic MALT lymphoma, the atypical lymphoid cells are small in size, with mildly irregular nucleoli, dense chromatin, and scant cytoplasm, without germinal center differentiation (**Figure 3G**) (64, 67). Because MALT lymphomas commonly involve the portal fields of the liver, lymphoepithelial lesions are always presented in the bile ducts, which is a typical accompanying feature of hepatic MALT lymphoma (68). In primary hepatic follicular lymphoma, it mainly presents as distinct follicles, formed by small- to intermediate-sized centrocytes, with germinal center differentiation (69). The presence of follicular meshworks is a vital feature in the diagnosis of follicular lymphoma (64). In primary hepatic Burkitt lymphoma, diffuse medium-sized lymphocytes, with distinct nucleoli, scant cytoplasm, and increased mitotic figures, form the lesions (64). A typical starry-sky appearance has been reported, which is formed by the dispersal of numerous tangible-body macrophages among malignant cells (62, 70).

Immunohistochemistry is always required for classification of lymphoma, which is important to obtain the correct diagnosis. Most PHLs are of B-cell origin, which are always positive for part of or all the B-cell markers, usually including CD19, CD20, and CD79a, and negative for CD3 (**Figures 2H**, **3H, I**) (71–74). However, different immunophenotypes have their own characteristics, which are essential for the classification of PHL. In DLCBL, they are often positive for CD45, PAX5, and BCL-2 with a high Ki-67 index (**Figure 2I**) (30, 75, 76). Patients with DLBCL are always subclassified into the germinal center B-cell (GCB) group, expressing CD10 and/or BCL-6 without MUM-1, and the non-GCB group, expressing MUM-1 without CD10 and BCL-6 (**Figures 2J–L**) (37, 64). In MALT lymphoma, they are also positive for IgM and CD21 and negative for CD5, CD10, and cyclin D1, with a low Ki-67 index (**Figures 3J–L**) (68, 77–79). In Burkitt lymphoma, they are also positive for monotypic surface IgM, CD10, BCL-6, and MYC and negative for BCL-2, with a high Ki-67 proliferative index, which is nearly 100% (64, 80). In follicular lymphoma, they are also positive for CD10, BCL-2, and BCL-6, with a low Ki-67 proliferative index. Immunostaining with CD21 and CD23 can highlight the follicular meshworks (64, 81, 82). The characteristics of pathology and immunophenotype of PHL are summarized in **Table 1**.

### Differential diagnosis

Due to non-special symptoms and imaging features, a number of liver diseases should be considered during differential diagnosis of PHL including hepatocellular carcinoma, metastatic disease of liver, cholangiocarcinoma, hepatitis, or systemic NHL with secondary hepatic involvement. Some clinical and imaging features are helpful in differentiating between these entities.

Hepatocellular carcinoma appears as marked enhancement in late arterial phase, which becomes both progressive washout of contrast compared to healthy liver tissue in portal venous or delayed phases (83). The “bulls-eye” sign is a characteristic feature of metastatic carcinoma of liver. It always appears as a thick rim-like enhancement in the arterial phase and a hypo-enhancement in portal venous and delayed phases (84). Patients with acute hepatitis appear to have a thickening of the gallbladder wall and hepatic periportal lucency, which can favor a diagnosis of acute hepatitis (85). SHL typically presents as multifocal or diffuse lesions along with extrahepatic involvements, which can be detected by CT, MRI, or PET/CT (86).

Although some features are useful clues in diagnosis, it is difficult to make a definite diagnosis without histological analysis, which is the gold standard of diagnosis.

### Prognosis and treatment

PHL with different infiltration types has a different prognosis. The 1-year and 3-year survival of patients with nodular infiltration are 70% and 57%, respectively, whereas those of patients with diffuse infiltration are 38% and 18%, indicating a poorer prognosis (87). In addition, the prognosis of indolent lymphoma, like follicular lymphoma and MALT lymphoma, is better than that of aggressive diseases like DLBCL, Burkitt lymphoma, and T-cell lymphoma (88). Major prognostic factors associated with survival are the large size of the tumor, high proliferation of cells, old age, systemic symptoms, unfavorable histologic subtype, disease stage, and complications (44, 68). An elevated level of LDH, β2 microglobulin, or serum calcium, as prognostic markers, suggests a worse prognosis (41).

Because of the rarity of PHL, no consensual recommendation for treatment has been issued. The choices of management for PHL contains surgery, chemotherapy, radiotherapy, or combinations of the above modalities (49). Liver transplantation has also been used in PHLs (68). The isolated case report and case series studies of PHL in the recent 10 years are summarized in **Supplementary Table 1**. As shown in **Table S1**, systemic combination chemotherapy is commonly used as the main therapeutic approach, which could usually achieve disease remission (89–91). Moreover, chemotherapy with CHOP-based regimens (cyclophosphamide, doxorubicin, vincristine, and prednisone) is the first-line treatment (92, 93). The combination of rituximab with conventional chemotherapy can prolong survival of PHL patients who are positive for CD20 (69, 94–96). The role of surgery is not clear, but some studies have found that surgical resection can offer a good outcome (97–102). Solitary lesions could be considered as the best candidates for hepatectomy and another indication for surgery is related to an uncertain diagnosis (30). In a review of 72 patients with PHL, Avlonitis et al. confirmed that surgical resection followed by adjuvant chemotherapy and radiotherapy is the optimal treatment for PHL. Patients treated with surgery and chemotherapy might have better prognosis than those treated with chemotherapy alone (38). However, due to the rarity of PHL, the definite role of surgery still needs to be further confirmed. Lymphomas also respond to radiotherapy, while radiotherapy is not as effective as chemotherapy, which is always used as an adjunct to chemotherapy (93, 103–106). Radiofrequency ablation (RFA), a new option for the treatment of PHL, has good efficacy in the short term. When the preoperative diagnosis is clear and the mass measures less than 2 cm, RFA can be selected. However, the effect of RFA in the long term needs more relevant cases (77).

## Primary biliary lymphoma

### Risk factors and pathogenesis

Due to the rarity of PBL, the exact etiological factor is obscure. Several studies found that half of all reported cases had gallstone, and this implies that PBL may be related to inflammation such as chronic cholecystitis or cholangitis associated with cholelithiasis or infected bile (107). Furthermore, mechanical irritation of gallstones is more responsible for the pathogenesis of PBL (108). PBL can also be found in patients with hepatitis virus infections and immunosuppression, such as HIV infection and organ transplantation (109, 110).

Whatever the specific cause of inflammation, cholecystitis or cholangitis induces lymphocyte migration to the mucosa of the biliary tract and prolonged lymphoid reactive proliferation, which is antigen-dependent, and accumulation in the site of inflammation, thus leading to irreversible chromosomal translocations, which can inhibit apoptosis and cause antigen-independent proliferation (111–113). Bisig et al. (108) detected a specific chromosomal translocation t (11, 18) (q21; q21) in primary biliary MALT lymphoma, leading to the expression of a transcript fusing the apoptosis inhibitor 2 (API2) gene to MALT1 gene. API2/MALT1 fusion can reduce the inhibition of API2 on apoptosis response to antigen stimulation, thus leading to MALT lymphoma of the biliary tract (108). Its production can also induce expression of BCL-10 and activation of the NF-κB pathway, leading to cell proliferation (**Figure 1**) (27). Another possible mechanism is that prolonged chronic inflammation causes irreversible genetic rearrangements, thus disabling the response of cells to IL-2 regulation, eventually helping in the development of MALT lymphoma (114).

In addition, another possible pathogenesis was reported by Angelopoulou and his colleagues. In PBL, the malignant transformation of the original clone could have occurred outside the biliary system with subsequent homing by an adhesion molecule mechanism (115).

### Epidemiology and clinical features

PBL is an extremely rare entity, which could be divided into intrahepatic bile duct and the extrahepatic biliary system. The clinical and radiological findings of lymphoma of the intrahepatic duct resemble those of hepatic lymphoma. Hence, we only discuss primary lymphoma of the extrahepatic biliary system here. Extrahepatic biliary non-Hodgkin’s lymphoma (EBNHL) constitutes 0.6% of malignant biliary tumors, including lymphoma of gallbladder and extrahepatic bile duct (8). Primary lymphoma of gallbladder has a higher prevalence than extrahepatic bile duct lymphoma. PBL can occur in various age groups and commonly affect elderly individuals with a mean age of 75.8 years old, with a slight female preponderance (11).

Patients with primary gallbladder lymphoma commonly present with symptoms of acute or chronic cholecystitis, and the most common symptom is upper right abdominal pain (11, 116). The common clinical manifestation of primary bile duct lymphoma is obstructive jaundice (8). Fever, night sweats, weight loss, nausea, and vomiting can also be present in these patients (117). The physical examination of these patients is often normal, while yellowing of skin and sclera with itching marks on the skin can be found in PBL patients with biliary obstruction (118). Slight tenderness in the upper right quadrant is also be observed in some cases.

The common abnormalities revealed by laboratory test results associated with PBLs include variably elevated bilirubin and liver enzyme levels, which suggest cholestasis (119). Interleukin (IL)-2 receptor, a serum marker of lymphoma, increases in patients with PBL (107, 120). Tumor markers are often within normal range, which can differentiate PBL from carcinoma of the biliary system, while mildly elevated CA19-9 can be observed in some cases (117). Full blood counts are usually normal at the early stage; however, the number of erythrocytes, leukocytes, and platelets might decrease when bone marrow or spleen is involved (8). In addition, other rare laboratory abnormalities include elevated serum and urine amylase level (121).

### Imaging

Radiological characteristics of PBL depend on their pathological classifications. Their imaging appearances can be divided into two morphological groups (1): a solid mass, seen in high-grade lymphomas, such as DLBCL; and (2) an irregular thickening in the gallbladder or bile duct wall, seen in low-grade lymphomas, such as MALT and follicular lymphoma (107, 111).

US is the modality of choice in the initial evaluation of gallbladder and biliary diseases (122). The common US features of primary gallbladder lymphoma present as thickening of the gallbladder wall or a soft tissue mass located in gallbladder with or without gallstone (107, 109), which has lower echo compared to gallbladder carcinoma (123). In addition, patients with primary bile duct lymphoma usually present with thickening of the bile duct or a hypoechoic mass with dilation of the proximal bile duct (124). The imaging features of endoscopic ultrasound (EUS) are similar to those of typical US.

**Figure 4:**
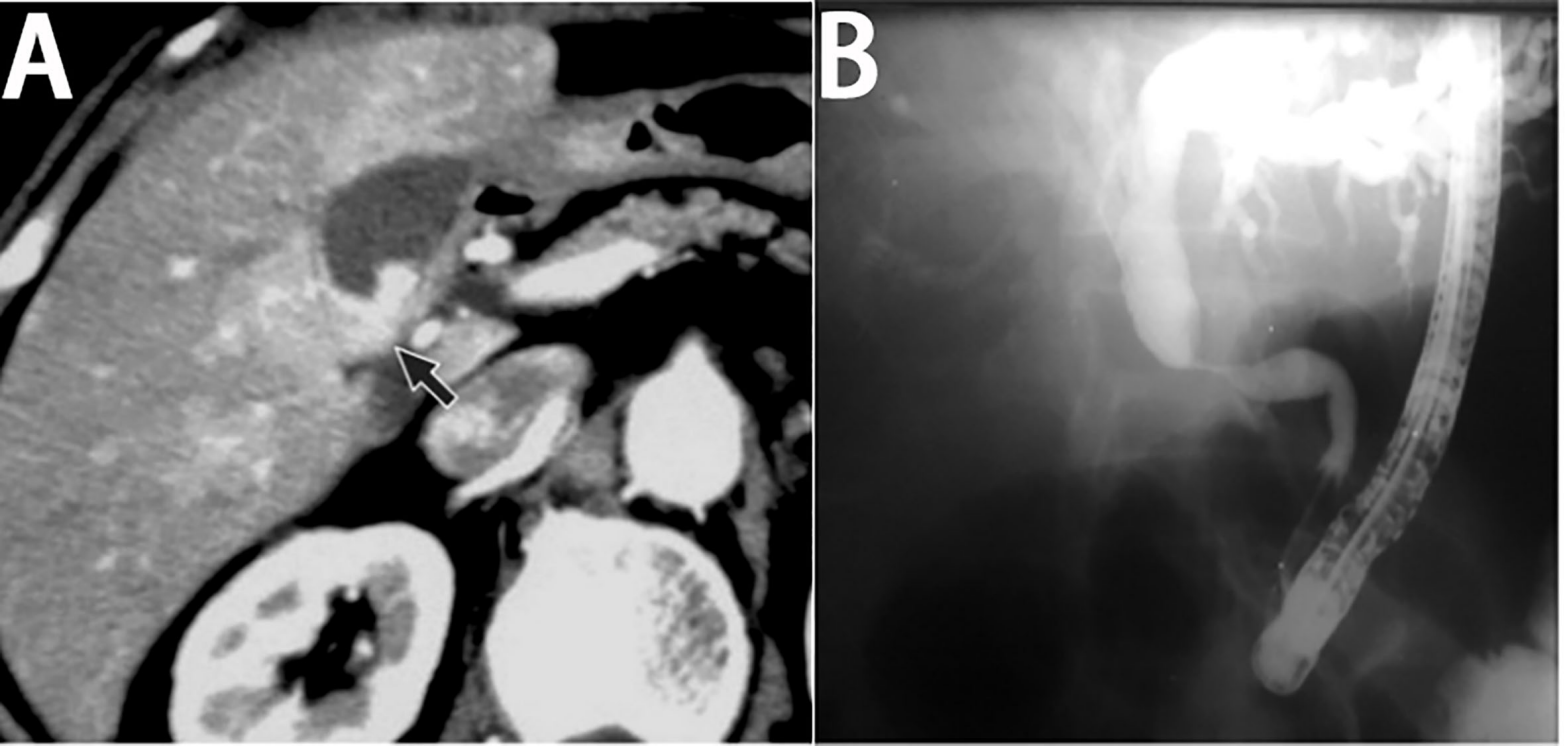
Imaging finding of primary biliary lymphoma. **(A)** On enhanced CT, the lesion of primary gallbladder lymphoma shows laminar enhancement on the mucosal surface of the gallbladder wall (black arrow). (with the permission of BIR publication). **(B)** ERCP of primary bile duct lymphoma shows a segmental luminal narrowing of mid common bile duct with marked dilation of proximal bile duct (with the permission of Elsevier).

The common CT feature of primary gallbladder lymphoma is a thickened gallbladder wall with intact mucosa and layered enhancement after administration of the contrast agent (**Figure 4A**) (107, 125). The other common CT feature presents as a focal mass confined in the gallbladder with slight enhancement (120, 126). Patients with primary bile duct lymphoma present with segmental circumferential wall thickening of the bile duct or a bile duct mass, with proximal bile duct dilatation and smooth mucosal layer (127).

On MRI, the gallbladder or bile ductal lesion shows lower and slightly higher signal intensity on the T1WI and T2WI, compared to the surrounding normal liver parenchyma (111, 127). Homogeneous enhancement of biliary lesion can be observed on contrast-enhanced images. The dilation of upstream bile duct can also be seen in most lymphomas of bile duct on MRI (128). MR cholangiopancreatography (MRCP) has rarely been applied to gallbladder lymphoma, and thus, the MRCP findings of gallbladder lymphoma are rarely reported. Moreover, MRCP features of primary bile duct lymphoma are similar to those of cholangiocarcinoma, commonly presenting as a segmental luminal narrowing of bile duct without mucosal irregularity and with dilation of the proximal bile duct (128). Endoscopic retrograde cholangiopancreatography (ERCP) can observe similar features (**Figure 4B**).

On PET/CT, the location and size of PBL are similar to other imaging modalities. The common imaging feature with PET/CT demonstrates hypermetabolic activity in gallbladder or bile duct without other metastatic areas (129, 130). PET/CT is a preferred choice for staging lymphoma and helpful for differentiating PBL from secondary lymphoma and other diseases, particularly cholecystitis.

### Pathology and immunohistochemistry

Like other lymphoma, adequate biopsy is needed for definitive diagnosis. Most PBL patients obtain pathologic specimens by surgery, while it can also be obtained under CT, EUS, or ERCP guidance (131).

Grossly, low-grade PBL presents as thickening of the bile duct and gallbladder with intact mucosal layer; however, in high-grade PBL, it shows a mass defined in the biliary system. Histologically, a dense and diffuse infiltration of atypical lymphoid cells in the gallbladder or bile duct wall, with intact mucosal layer and occasional lymphoid follicles, could be observed in patients with low-grade lymphoma [127, 132], whereas in patients with high-grade lymphoma, it demonstrated diffuse infiltration of large-sized lymphocytes with prominent nucleoli and abundant mitoses [36].

As summarized in **Table 3**, the most common subtypes of primary lymphoma of gallbladder or bile duct are DLBCL and MALT, followed by follicular lymphoma and B-lymphoblastic lymphoma (11). The immunohistochemical features and molecular findings of different subtypes of PBL are summarized in **Table 3**, which are similar to those of PHL (83, 112, 123–128, 132, 133).

### Differential diagnosis

PBL is a rare disorder that can be present as a focal mass or a thickening in the gallbladder or bile duct wall and imitate the characteristics of biliary tumors such as adenocarcinoma or inflammatory process such as sclerosing cholangitis and cholecystitis (119).

Patients with cholangiocarcinoma presented as concentric or eccentric wall thickening without intact mucosa, resulting in varying degrees of luminal stenosis (134). Gallbladder carcinoma always appears as a mass or thickening of the gallbladder wall, with disruption of the mucosal layer (135). Primary sclerosing cholangitis (PSC), associated with inflammatory bowel disease (IBD), presents as multifocal biliary strictures (136). Radiographical findings of cholecystitis show a thickened gallbladder wall (>4 mm), often accompanied by gallstones and pericholecystic fluid (137). Besides imaging features, patient’s disease history, physical examination, and laboratory studies are essential for differentiation diagnosis (122).

The prognosis and treatment of PBL are different from other biliary diseases, and the proper diagnosis of the disease is important. The histological analysis is indispensable for a correct diagnosis.

### Prognosis and treatment

PBL has a better prognosis than cholangiocarcinoma and gallbladder carcinoma (118). Like other NHLs, the prognosis of PBL may be associated with age, tumor stage, subtype, and treatment. Due to lack of sufficient case series, this association has not been confirmed; more studies are needed to prove this.

Due to the low incidence of PBL, there is no consensus on the optimal treatment. As shown in **Table S2**, the management consists of surgery, chemotherapy, radiotherapy, or a combination of all. Because of the difficulty of acquiring pathological diagnosis, surgical resection is commonly performed in most PBL patients (**Table S2**) (120). Surgery can also be a therapeutic option for patients with complicating biliary obstruction, or who fail to respond to chemotherapy (119). Surgical intervention is proven curative in many cases (121, 138). Chemotherapy is considered as the predominant management modality and an integral part of the postoperative treatment (124). Radiotherapy is often considered an adjunct to chemotherapy, which might increase the survival of patients with residue after chemotherapy (128). From reviewing previous PBL case studies, surgical resection, combined with chemotherapy with or without radiotherapy may be a treatment regimen for improved survival rate, which need further studies to confirm.

## Primary pancreatic lymphoma

### Risk factors and pathogenesis

Due to the rarity of PPL, the consensus of pathogenesis is still not issued. It is commonly associated with immunosuppression, related to HIV infection or solid organ transplantation (139). A few studies have implied that HCV or HBV infection is associated with PPL (140, 141).

However, to our knowledge, HBV and HCV are hepatotropic and are main etiologies of liver cancer. The possible reasons that hepatitis virus can cause pancreatic damage are the proximity of the liver to the pancreas and shared blood vessels and ducts. The possibility is further supported by findings of hepatitis B surface antigen (HBsAg) in pancreatic juice and hepatitis virus replication in pancreatic cells among patients with HBV infection (142, 143).

Like PHL, HBV or HCV infection may indirectly give rise to inflammation-associated lymphomagenesis in the pancreas. It may induce cytokine and cytokine growth, perhaps affecting various genes and causing proliferation of lymphoid cells (140). Moreover, it may also reduce the threshold of antigen response or result in DNA mutations by binding to surface receptors of B lymphocytes, thus leading to lymphoproliferation (141).

### Epidemiology and clinical features

PPL is a rare disease and accounts for approximately 1% of extranodal lymphoma and 0.5% of pancreatic cancers (9). PPL commonly affects middle-age individuals with a median age of 53 years old and has a male predilection (male:female ratio of 1.5:1) (12).

Clinical features of PPL are non-specific and include epigastric pain, abdominal mass, weight loss, obstructive jaundice (144–146), intestinal obstruction, and rarely acute pancreatitis (147), which are similar to those of pancreatic adenocarcinoma (148, 149). However, the typical symptoms of NHL, such as fever, chills, and night sweats, are rare in PPL (150). A physical examination was significant for epigastric tenderness, abdominal mass, and jaundice, but not for organomegaly or lymphadenopathy.

The tumor marker levels in PPL patients, such as CA19-9 and CEA, are commonly normal or slightly elevated in the case of biliary obstruction, which are apparently increased in most pancreatic adenocarcinoma patients (151). An elevated LDH level and serum beta-2-microglobulin are often presented in patients with pancreatic lymphoma. Although their elevations are not necessarily required for the diagnosis of PPL, they are useful markers, high levels of which are indicators of a poor prognosis for patients with PPL (152). Elevated liver enzymes and bilirubin levels are found in PPL patients with biliary obstruction.

### Imaging

Radiologically, the lesions of PPL appear as focal and well-defined lesions or diffuse infiltration of pancreas (153), as summarized in **Table 2**. The tumor is most commonly located in the head of the pancreas, though it can be found in other portions of the pancreas, such as the body and tail (149, 154). PPL almost presents as bulky masses with a median size of 7.9 cm (155).

The most common US finding is a bulky homogeneous hypoechoic mass confined to the pancreas, with or without dilatation of bile ducts. Peripancreatic vessels are encompassed by mass, but are always not infiltrated, which can be distinguished from pancreatic adenocarcinoma (12, 156). The appearances of EUS are consistent with those of typical US; however, it has a higher sensitivity (157).

Boninsegna et al. found that PPLs have the following common CT features: a large and hypo-attenuation mass with mild enhancement (**Figures 6A–C**), peri-pancreatic fat stranding, vessel encasement without infiltration, rare pancreatic duct dilatation, and absence of necrosis (158, 159). These findings are in agreement with some studies reported by other teams (12, 160, 161). The “sandwich sign”, in which a mesenteric mass surrounds the mesenteric vessels, without vascular infiltration, is frequently observed in patients with PPL (161, 162).

**Figure 5:**
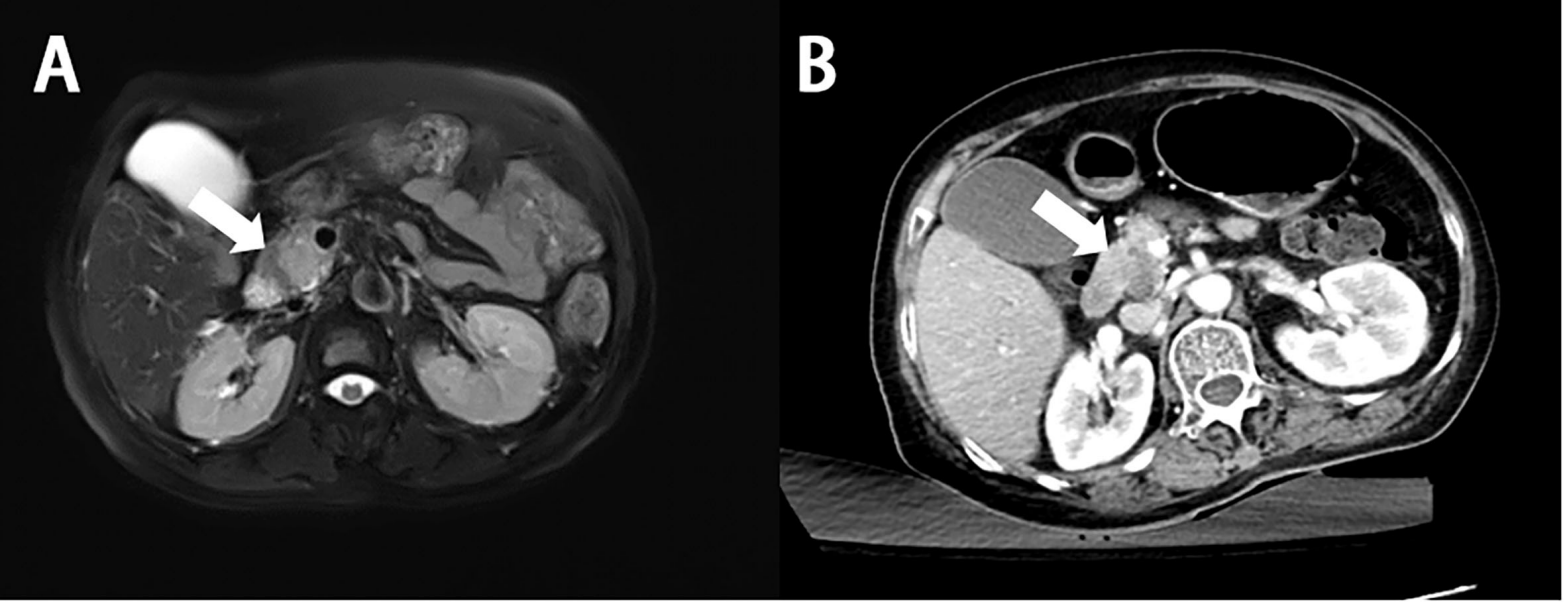
Imaging finding of primary pancreatic diffuse large B-cell lymphoma. **(A)** MR shows a lesion with hyperintense signal on the T2-weighted images (white arrow). **(B)** On enhanced CT, the lesion shows a mild enhancement, lower extent than the normal parenchyma (white arrow).

The MRI characteristics of PPL appear as a bulk lesion with mild enhancement, without significant pancreatic duct dilatation, which has a lower signal intensity on T1WI and a higher signal on T2WI (**Figures 5A, B**) (12, 163, 164). Unlike CT, MRI shows a slightly heterogeneous character of the lesion, especially on T2WI (165). In some cases, PPL may present with diffuse enlargement of pancreas, mimicking pancreatitis (166, 167). Occasionally, PPL may initially present only as acute pancreatitis and can be diagnosed only on follow-up imaging (167). MPD dilatation can rarely be found in PPL patients. However, mildly upstream MPD dilatation is another feature of PPL with diffuse pancreatic enlargement (165).

The most common PET/CT feature is a solitary hypermetabolic lesion in the pancreas, with SUVmax ranging from 7.4 to 26.5 (the mean SUVmax is 13.2) (168–171).

### Pathology and immunohistochemistry

Pancreatic tissue can be obtained by percutaneous/endoscopic FNA or biopsy (172), exploratory laparotomy, or resection surgery. EUS-guided FNA or biopsy is an optimal method to obtain preoperative diagnosis (132, 157, 173–175).

As confirmed in **Table S3**, the most predominant PPL subtype is DLBCL (occupying nearly 77%), followed by follicular lymphoma (occupying 14%) (155). Furthermore, Burkitt lymphoma, small lymphocytic and T-cell lymphoma, or Hodgkin’s lymphoma can also be present in PPL (176–180).

**Figure 6:**
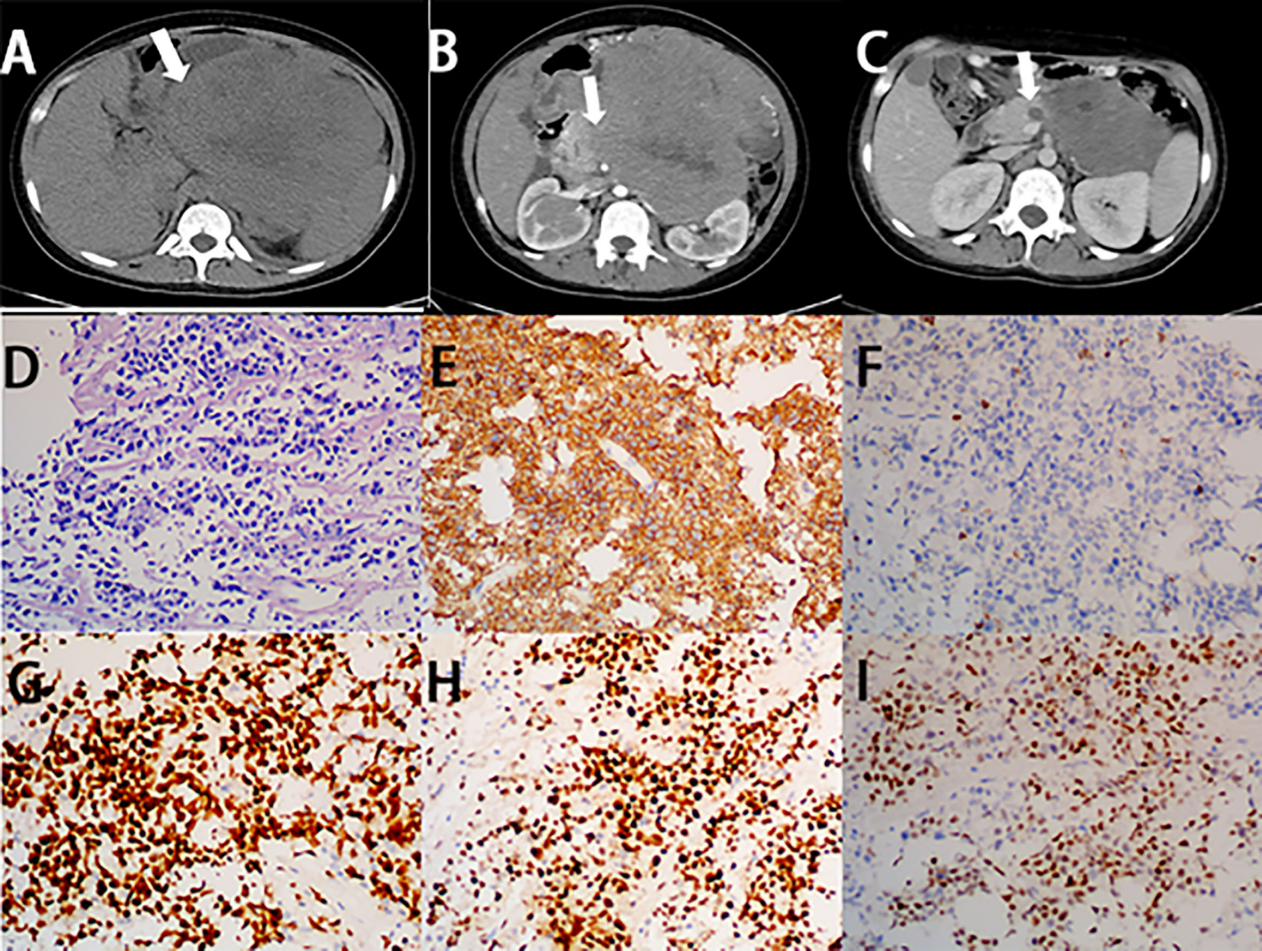
Imaging finding and histology of primary pancreatic Burkitt lymphoma. **(A)** Unenhanced CT of PPL shows a bulky and heterogeneous mass, with irregular margin (white arrow). **(B)** On the horizontal plane of enhanced CT, the lesion shows mild enhancement (white arrow). **(C)** After three cycles of chemotherapy, the size of the lesion significantly decreased. **(D)** A diffuse infiltrate of medium-sized lymphocytes replaces the pancreas parenchyma with starry-sky appearance (H&E stain, 20×). **(E)** Lymphocytes are positive for CD20 (CD20 immunostain, 20×). **(F)** Lymphocytes are negative for CD3 (CD3 immunostain, 20×). **(G)** The Ki-67 proliferative index is high in the lymphocytes (Ki-67 immunostain, 20×). **(H)** Lymphocytes are positive for BCL-6 (BCL-6 immunostain, 20×). **(I)** Part of lymphocytes are positive for Myc (Myc immunostain, 20×).

The specific pathologic and immunohistochemical findings of PPL are demonstrated in **Table 2** and in **Figures 6D–I** (181–194).

### Differential diagnosis

The clinical manifestations of PPL may mimic those of other neoplastic or inflammatory pancreatic diseases, such as pancreatic adenocarcinoma, pancreatic neuroendocrine tumor, acute pancreatitis, and autoimmune pancreatitis (AIP) (195). Although PPL has overlapped symptoms and imaging findings with other pancreatic diseases, some characteristic findings can help in the diagnosis of PPL.

In contrast to PPL, pancreatic ductal dilatation, and ductal and peripancreatic vascular invasion were frequently shown in pancreatic adenocarcinoma (196, 197).

Both PPL and AIP might appear as diffuse enlargement, a focal lesion, or multifocal lesions, making differential diagnosis difficult. Ishigami et al. (166) observed 8 patients with pancreatic lymphoma and 21 patients with AIP to identify the point of imaging discrimination for the two diseases. They concluded that patients with AIP present with delayed enhancement with a capsule-like rim on CT and MRI, which was absent in the PPL imaging feature (166, 198). In addition, elevated serum c-globulin levels, particularly immunoglobulin G, can be observed in almost all of the patients with AIP, but not in PPL patients (199).

Even if some findings can help with the diagnosis, histological analysis is still required for a definitive diagnosis.

### Prognosis and treatment

The prognosis for PPL is much better than that of other pancreatic malignant tumors. A cohort study showed a median overall survival of 53 months (200). Patients with advanced age have a worse overall survival. Similarly, patients with stage IV or aggressive subtype have a worse prognosis, with a median survival of only 13 months, whereas those with an earlier stage have a longer survival of 80 months. Undergoing chemotherapy is also significantly associated with better overall survival, while location of tumor, race, and sex are not associated with overall survival (200).

The rarity of PPL patients makes it difficult to draw a definite conclusion about the optimal treatment, which is mainly determined by the histological subtype. As summarized in **Table S3**, the strategy for PPL includes surgery, chemotherapy, radiotherapy, or a combination of all (9). Considering that the pathology of most PPL patients is DLBCL, chemotherapy is the standard treatment (12, 155, 201). The most commonly used regimen is CHOP or R-CHOP (176). The majority of patients with PPL receiving only chemotherapy can achieve long-term disease remission (12, 148, 149, 163, 202). Vijungco et al. (203) found that early-stage NHL patients treated with radiotherapy alone have a high overall cure rate. However, in the last decade, radiotherapy has often been used as an adjunct to chemotherapy, rather than used alone (144, 151, 175, 188, 204). Some studies demonstrated that patients treated with chemotherapy with radiotherapy had a high overall response rate (144, 188). However, the role of radiotherapy has also not yet been well defined (204). Surgical intervention is not adopted as the primary treatment for PPL, which is considered when the diagnosis of a mass in pancreas remains uncertain or patients present symptoms caused by obstruction of the biliary tract (155). Behrns et al. (205) reported that the combination of surgery and chemotherapy has a better survival benefit than chemotherapy alone. Interestingly, a study by Facchinelli et al. (188) showed different results. Thus, surgical intervention remains controversial and needs to clarify its benefits through numerous PPL study series (188).

## Conclusion

**Figure 7:**
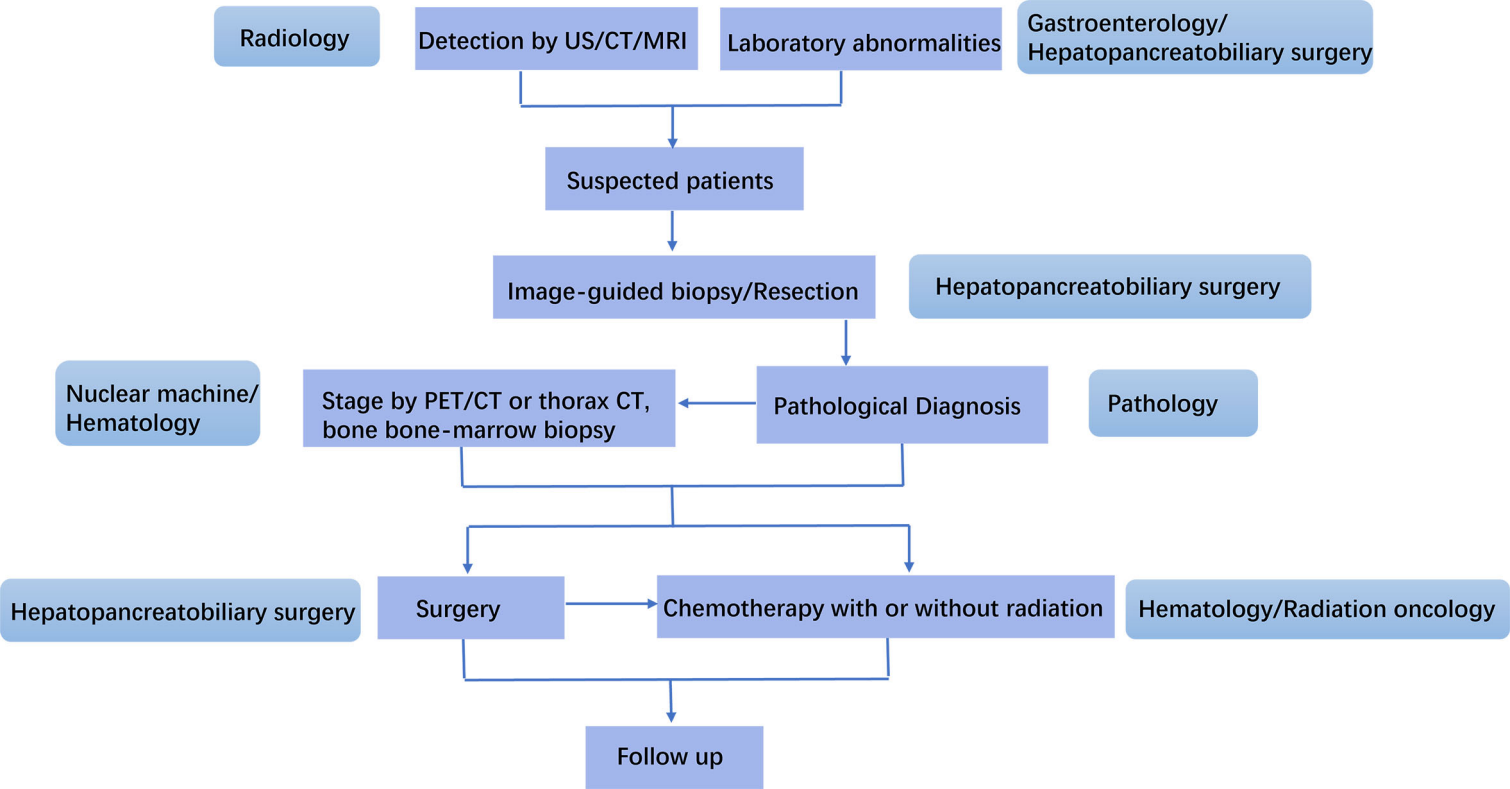
Management schedule for an experienced MDT for primary hepatopancreatobiliary lymphoma (PHPBL). Patients with characteristic laboratory abnormality are suspected as PHPBL after imaging finding of HPB mass. They will receive image-guided biopsy or resection. The pathological diagnosis will be made by the Department of Pathology. PET/CT or thorax CT, and bone/bone marrow biopsy are used to lymphoma stage. After that, the precise treatment will be discussed by hepatopancreatobiliary surgeons, hematologists, and radiation oncologists. After the treatment, the follow-up of patients will be made.

PHPBL often overlaps with other diseases of hepatobiliary and pancreas in clinical and radiological features, resulting in misdiagnosis and delayed treatment. Since early diagnosis depends on the alertness of clinicians, mainly radiologists, gastroenterologists, and hepatopancreatobiliary surgeons, it is important for them to know more clinical and imaging features of PHPBL, in order to obtain a proper diagnosis and management. Clinical manifestation, imaging findings, and laboratory studies could provide helpful clues for diagnosis, whereas histological analysis is the gold standard for accurate diagnosis and subtype analysis. Due to the rarity of the disease, there is no consensus on treatment options. The optimal therapeutic choice, including surgery, chemotherapy, radiotherapy, immunotherapy, or other treatments, either alone or in combination, needs further investigation. The management of PHPBL needs an experienced multidisciplinary team, involving radiologists, gastroenterologists, hepatopancreatobiliary surgeons, pathologists, hematologists, and radiation oncologists, to provide individualized therapy and better prognosis (**Figure 7**).

## Author contributions

Conceptualization, BZ. Literature search, QW, YL, and ZS. Investigation, KW and XZ. Project administration, BZ and SW. Writing—original draft, QW. Data curation, KW, XZ, and SW. Formal analysis, YL and ZS. Writing—review and editing, BZ. All authors have read and agreed to the published version of the manuscript.

## Funding

This review was funded by grants from the National Natural Science Foundation of China (No. 81570698).

## Conflict of interest

The authors declare that the research was conducted in the absence of any commercial or financial relationships that could be construed as a potential conflict of interest.

## Publisher’s note

All claims expressed in this article are solely those of the authors and do not necessarily represent those of their affiliated organizations, or those of the publisher, the editors and the reviewers. Any product that may be evaluated in this article, or claim that may be made by its manufacturer, is not guaranteed or endorsed by the publisher.
