# Supplementary material for: Primary hepatopancreatobiliary lymphoma: Pathogenesis, diagnosis, and management
Source: Front Oncol. 2022 Aug 30;12:951062. doi: 10.3389/fonc.2022.951062 (PMC9469986; doi:10.3389/fonc.2022.951062)
Supplement: Supplementary file 1 [file DataSheet_1.docx]

**Table S1: Case series or isolated case reports published in the field of PHL the last decade.**

| Reference | Number of cases | Age/sex | histotype | Tumor lesion | treatment | Follow-up |
| --- | --- | --- | --- | --- | --- | --- |
| Myoteri D et al [37] | 1 | 70/M | DLBCL | Multiple lesions | Chemotherapy(R-CHOP) | 18 months/in remission  Recurrence after 3 years |
| Wang L et al [57] | 1 | 29/M | DLBCL | Multiple lesions | Surgery+ RFA+ Chemotherapy  (R-CHOP) | 36 months/NED |
| Pan B et al [56] | 1 | 55/F | DLBCL | A solid lesion | Surgery | 25 months/NED |
| Mehta N et al [49] | 1 | 55/F | DLBCL | A solid lesion | Surgery + Chemotherapy  (R-CHOP) | NA |
| Lu Q et al [48] | 29 | 52(11-72)  M/F 19/10 | DLBCL 17  MALT 6  FL 1  TCL 5 | Single lesion 15  Multiple lesions 9  Diffuse 5 | Surgery or Chemotherapy | Diffuse: 12 months/NED 2  Died 12 days 1  Died 42 days 1  1 lost in follow-up  Nodule:  Surgery 12 months/NED  Chemotherapy 9 months/NED  4 lost in follow-up |
| Forghani F et al [44] | 1 | 67/M | DLBCL | Single lesion | Chemotherapy(R-CHOP) | in remission |
| Peng Y et al [10] | 10 | 50  M/F 2.3:1 | DLBCL 8  BL 1  TCL 1 | Single lesion 3  Multiple lesions 7 | Chemotherapy 8  Radiation 1  No therapy 1 | Mean follow-up duration: 50 months |
| Haefliger S et al [18] | 1 | 69/M | MALT | Single lesion | Chemotherapy(R-CHOP) | 6 months/PR |
| Abdelrahim WE et al [41] | 1 | 19/F | DLBCL | Single lesion | Surgery + Chemotherapy  (R-CHOP) | 24 months/NED |
| Yasuda T et al [98] | 1 | 54/F | MALT | Multiple lesions | Surgery | 12 months/NED |
| Choi S et al [59] | 1 | 70/M | MALT | Multiple lesions | Surgery | 8 months/NED |
| Xie H et al [99] | 1 | 73/M | MALT | Single lesion | Surgery | 6 months/NED |
| Muttillo EM et al [23] | 2 | 43/M  68 | DLBCL | Multiple lesions  Multiple lesions | Chemotherapy(R-CHOP)  Chemotherapy(R-CHOP) | 72 months/NED  NED |
| Mrabet S et al [14] | 1 | 52/NA | DLBCL | Multiple lesions | Chemotherapy(R-CHOP) | PR |
| Xu Z et al [77] | 1 | 63/F | MALT | Single lesion | RFA | 12 months/NED |
| Ali AM et al [39] | 1 | 82/F | DLBCL | Multiple lesions | Chemotherapy(R-CHOP) | NA |
| Martínez DTE et al [80] | 1 | 67/M | an intermediate type between DLBCL and BL | Multiple lesions | Chemotherapy(R-CHOP) | 18 months/NED |
| Zentar A et al [71] | 1 | 65/F | DLBCL | Single lesion | Surgery+ Chemotherapy (CHOP) | 24 months/NED |
| Imrani K et al [72] | 1 | 52/F | BCL | Multiple lesions | Chemotherapy | in remission |
| Kawakami H et al [95] | 1 | 78/M | DLBCL | Single lesion | Chemotherapy(R-CHOP) | NED |
| Liu Y et al [93] | 1 | 61/M | DLBCL | Multiple lesions | Chemotherapy (CHOP) | 24 months/NED |
| Xu J et al [81] | 1 | 53/F | FL | Multiple lesions | Surgery+ MWA+ Chemotherapy  (R-CHOP) | 24 months/NED |
| Yu JJ et al [100] | 1 | 34/M | DLBCL | Single lesion | Surgery + Chemotherapy  (R-CHOP) | 12 months/NED |
| Resende V et al [104] | 1 | 69/M | DLBCL | Single lesion | Chemotherapy(R-CHOP) +radiotherapy | 24 months/PR |
| Dantas E et al [96] | 1 | 65/M | DLBCL | Multiple lesions | Chemotherapy(R-CHOP) | Die of complication |
| Ozaki K et al [94] | 1 | 73/F | DLBCL | Single lesion | Chemotherapy (CHOP) +  radiotherapy | 36 months/NED |
| Williams MO et al [82] | 1 | 71/M | FL | Diffuse | Chemotherapy | Died 40 days after admission |
| Yu YD et al [73] | 1 | 38/M | MALT | Multiple lesions | Surgery+ Chemotherapy (CHOP) | 15 months/NED |
| Scucchi L et al [19] | 1 | 85/M | FL | Single lesion | Immunotherapy (rituximab) | Die of ischemic heart attack |
| Ul Haq F et al [66] | 1 | 55/M | DLBCL | Single lesion | Surgery + Chemotherapy | NA |
| Yaka M et al [74] | 1 | 58/M | DLBCL | Single lesion | Surgery+ Chemotherapy (R-CHOP) | Die of a complication of chemotherapy |
| Xing AY et al [65] | 7 | 61(39-77)  M/F 4/3 | DLBCL | Single lesion 6  Multiple lesions 1 | Chemotherapy (R-CHOP) 4  NA 3 | Survivors: 51.2 months 5  Died 1 month after diagnosis 1  NA 1 |
| Zaimi Y et al [17] | 1 | 58/M | DLBCL | Multiple lesions | Chemotherapy (R-CHOP) | PR |
| Nishikawa K et AL [58] | 1 | 85/M | DLBCL | Multiple lesions | No treatment | Died 26 days after diagnosis |
| Panda SS et al [42] | 1 | 75/F | MALT | Single lesion | Immunotherapy (rituximab) changed to Chemotherapy (R-CHOP) due to PD | SD |
| El Nouwar R et al [32] | 1 | 55/F | DLBCL | Diffuse | No treatment | Die 2 days after diagnosis |
| Kaneko R et al [33] | 1 | 73/M | DLBCL | Single lesion | Chemotherapy (R-CHOP) | 12 months/NED |
| Grewe S et al [67] | 1 | 78/F | MALT | Single lesion | Surgery | NA |
| Betianu CI et al [46] | 1 | 47/F | MALT | Single lesion | Surgery+ Chemotherapy (R-CHOP) | 9 months/NED |
| Park JI et al [101] | 1 | 65/M | DLBCL | Single lesion | Surgery+ Chemotherapy (R-CHOP) | 60 months/NED |
| Farag F et al [34] | 1 | 82/F | DLBCL | Diffuse | No treatment | Die of PD |
| Muthukrishnan S et L [60] | 1 | 55/M | HL | Single lesion | Surgery+ Chemotherapy (ABVD) | NED |
| Park JE et al [20] | 1 | 75/F | DLBCL | Single lesion | Chemotherapy (R-CHOP) | 24 months/NED |
| Bohlok A et al [79] | 1 | 68/M | MALT | Single lesion | Surgery | NA |
| Hu HJ et al [61] | 1 | 59/M | TCL | Single lesion | Surgery+ Chemotherapy (CHOP) + radiotherapy | 12 months/NED |
| Takei D et al [75] | 1 | 65/F | DLBCL | Single lesion | Surgery | 12 months/NED |
| Modi G et al [62] | 1 | 21/F | BL | Multiple lesions | Chemotherapy | NED |
| Wu GB et al [47] | 1 | 56/M | MALT | Single lesion | Surgery+ Chemotherapy (CHOP) | NED |
| Bouliaris K et L [102] | 1 | 57/F | DLBCL | Single lesion | Surgery+ Chemotherapy (R-CHOP) | 12 months/NED |
| Patel TS et al [36] | 1 | 60/M | DLBCL | Single lesion | Chemotherapy (CHOP) | NA |
| Hsu A et al [40] | 1 | 65/F | DLBCL | Single lesion | Chemotherapy (R-CHOP) | In remission |
| Nagata S et al [35] | 1 | 74/M | MALT | Single lesion | Surgery | 24 months/NED |
| Widjaja D et al [91] | 1 | 32/M | DLBCL | Multiple lesions | Chemotherapy | NED |
| Valladolid G et al [76] | 1 | 76/F | DLBCL | Single lesion | Surgery | NED |
| Zafar MS et al [92] | 1 | 68/F | DLBCL | Single lesion | Chemotherapy (R-CHOP) | 18 months/NED |
| Do TD et al [51] | 1 | 36/M | DLBCL | Multiple lesions | Chemotherapy (R-CHOP) | NA |
| Laroia ST et al [97] | 1 | 67/M | DLBCL | Multiple lesions | Chemotherapy (R-CHOP) | 12 months/NED |
| Zhang KJ et al [107] | 1 | 56/M | DLBCL | Multiple lesions | Chemotherapy + radiotherapy | ＞36 months/NED |
| Albano D et al [106] | 5 | 66(54-80)  M/F 4/1 | MALT | Single lesion/ Multiple lesions 4/1 | Chemotherapy 3  Chemotherapy+radiotherapy1  Radiotherapy 1 | NED |
| Steller EJ et al [103] | 1 | 59/F | DLBCL | Single lesion | Surgery+ Chemotherapy (R-CHOP) | 24 months/NED |
| Tammana VS et al [105] | 1 | 45/M | DLBCL | Multiple lesions | Chemotherapy+ radiotherapy | NED |

*MALT: mucosa-associated lymphoid tissue lymphoma; FL: follicular lymphoma; DLBCL: diffuse large B cell lymphoma; TCL: T-cell lymphoma; BCL: B-cell lymphoma; BL: Burkitt lymphoma; HL: Hodgkin's lymphoma; RFA: radiofrequency ablation; PR: partial regression; MWA: microwave ablation; NED: no evidence of disease; NA: no accessed; SD: stable disease; R-CHOP: rituximab, cyclophosphamide, doxorubicin, vincristine, and prednisone; ABVD: Adriamycin, bleomycin, vinblastine and dacarbazine.

**Table S2: Case series or isolated case reports published in the field of PBL the last decades.**

| Reference | Number of cases | Age/sex | histotype | Tumor lesion | Treatment | Follow-up |
| --- | --- | --- | --- | --- | --- | --- |
| Shah KSV et al [122] | 1 | 80/M | MALT | Gallbladder | Surgery | 12 months/NED |
| Elbanna KY et al [120] | 1 | 71/M | FL | Common bile duct | Surgery | NA |
| Durham C et al [131] | 1 | 61/M | HGBCL | Common bile duct | Chemotherapy(R-CHOP) | NA |
| So A et al [130] | 1 | 24/M | HGBCL | Gallbladder | Surgery+ Chemotherapy(R-CHOP) | 4 months/NED |
| Joo YE et al [129] | 1 | 21/F | DLBCL | Common bile duct | Surgery+ Chemotherapy (CHOP)+Radiotherapy | 17 months/NED |
| Yoon MA et al [128] | 1 | 62/M | MALT | Extrahepatic bile duct | Surgery | NA |
| Kato H et al [121] | 1 | 55/F | DLBCL | Gallbladder | Surgery+ Chemotherapy(R-CHOP) | 38 months/NED |
| Wong DL et al [125] | 1 | 32/M | DLBCL | Common bile duct | Surgery+ Chemotherapy(R-CHOP) | in remission |
| Ono A et al [108] | 1 | 78/F | FL | Gallbladder | Surgery+ Chemotherapy | in remission |
| Batur A et al [110] | 1 | 60/NA | NHL | Gallbladder | Surgery | Die of cirrhosis |
| Mikail C et al [118] | 1 | 58/F | HGBCL | Extrahepatic bile duct | Surgery+ Chemotherapy(R-CHOP) | 12 months/NED |
| Psarras K et al [112] | 1 | 85/M | SLL | Gallbladder | Surgery | 12 months/NED |
| Gao F et al [8] | 1 | NA/M | NK/T lymphoma | Gallbladder | Surgery | Died 5 days after surgery |
| Zakaria A et al [119] | 1 | 57/M | DLBCL | Common bile duct | Surgery + Chemotherapy(R-CVP) | 60 months/NED |
| Park YK et al [113] | 1 | 86/F | MALT | Common bile duct | Surgery | 12 months/NED |
| Cho YH et al [115] | 1 | 79/M | MALT | Common bile duct | Surgery | NA |
| Shito M et al [133] | 1 | 71/M | MALT | main hepatic duct | Surgery+ Chemotherapy (CHOP) | 45 months/NED |
| Dote H et al [134] | 1 | 63/M | DLBCL | Common bile duct | Surgery+ Chemotherapy(R-CHOP) | 8 months/NED |
| Das K et al [111] | 2 | 36/M  51/M | DLBCL  DLBCL | main hepatic duct  main hepatic duct | Surgery+ Chemotherapy (CHOP)  Chemotherapy(R-CHOP) | 68 months/NED  18 months/NED |
| Pezzuto R et al [117] | 1 | 63/F | SLL | Gallbladder | Surgery | NED |
| Karia M et al [126] | 1 | 81/M | MALT | Gallbladder | Surgery | NED |
| Acharya V et al [127] |  | 75/F | FL | Gallbladder | Surgery | 6 months/NED |
| Yu SC [139] | 1 | 40/F | DLBCL | Common bile duct | Surgery+ Chemotherapy | 10 months/NED |

* MALT: mucosa-associated lymphoid tissue; FL: follicular lymphoma; DLBCL: diffuse large B cell lymphoma; SLL: small lymphocytic lymphoma; HL: Hodgkin lymphoma; HGBCL: high-grade B-cell lymphoma; NED: no evidence of disease; NA: no accessed; R-CHOP: rituximab, cyclophosphamide, doxorubicin, vincristine, and prednisone.

**Table S3: Case series or isolated case reports published in the field of PPL the last decade**.

| Reference | Number of cases | Age/sex | histotype | Tumor lesion | Treatment | Follow-up |
| --- | --- | --- | --- | --- | --- | --- |
| Battula N et al [206] | 1 | 63/M | DLBCL | Single mass/head | Surgery+ chemotherapy（CHOP） | NA |
| Shahar KH et al [205] | 1 | 70/F | NA | Single mass/head | chemotherapy（CHOP）+ radiotherapy | 21 months/NED |
| Facchinelli D et al [189] | 39 | 57(15-79)  M/F 22/17 | DLBCL 27  HGBCL 4  FL 4  BL 1  HL 2  TCL 1 | Head/other sites  28/11 | Chemotherapy 22  chemotherapy + radiotherapy 6  surgery + chemotherapy 3  surgery 1  no treatment 2 | Chemotherapy  CR 15/22  PR 5/22  SD 2/22  chemotherapy + radiotherapy  CR 3/6  PR 2/6  PD 1/6  5 lost in follow-up |
| Savari O et al [184] | 2 | 53/F  55/F | DLBCL  DLBCL | Multiple masses  /head  Single mass/tail | NA  chemoimmunotherapy | Died 5 weeks after diagnosis  14 months/ in remission |
| Yu LL et al [177] | 2 | 32/M  62/M | DLBCL  DLBCL | Single mass/head  Single mass/tail | Surgery+ chemotherapy（CHOP）  chemotherapy（CHOP） | 16 months/NED  NED |
| Lin H et al [152] | 6 | 46(16-65)  M/F 5/1 | BCL | Head/body and tail/  Whole 3/2/1 | Surgery + chemotherapy 4  chemotherapy+ radiotherapy 2 | NED  Died 49 months after diagnosis  Died 37 months after diagnosis  Died 2 weeks after diagnosis  2 lost to follow-up |
| Shirai Y et al [168] | 1 | 71/F | FL | Single mass/body | Surgery+ chemotherapy | NA |
| Hughes B et al [163] | 1 | 54/F | DLBCL | Single mass/head | chemotherapy（R-CHOP） | NED |
| Liakakos T et al [154] | 1 | 65/M | DLBCL | Single mass/head | Surgery+ chemotherapy（CHOP） | 21 months/NED |
| Sadot E et al [156] | 44 | 62.5(15–85)  M/F 24/20 | DLBCL 34  FL 6  HGBCL 3  BL 1 | Head/body and tail  22/22 | Chemotherapy 25  chemotherapy + radiation 3  surgical resection+ chemotherapy 3  surgical bypass+ chemotherapy 5  surgical resection 2  no treatment 2 | Chemotherapy 51 months  Surgical bypass+ chemotherapy 164.8 months  Surgical resection+ chemotherapy  85 months/SD  died 23 months after diagnosis  34 months/NED  Surgical resection  died 13 days after surgery  died 8 months after surgery  5 lost in follow-up |
| Tikue A et al [149] | 1 | 71/M | DLBCL | Single mass/head | chemotherapy（R-CHOP） | 18 months/NED |
| Alzerwi NAN et al [150] | 1 | 53/F | NHL | Single mass/head | chemotherapy（R-CHOP） | 12 months/NED |
| Shnitser A et al [188] | 1 | 19/F | DLBCL | Multiple masses/  Head and tail | surgery | NED |
| Dunphy L et al [9] | 1 | 52/M | DLBCL | Single mass/head | Chemotherapy+ radiotherapy | Died 5 months after diagnosis |
| Bhagat VH et al [111] | 1 | NA/F | BL | Single mass/head | Chemotherapy | Die of complications |
| Sallapan S et al [193] | 1 | 75/M | FL | Single mass/head | surgery | NA |
| Qiu T et al [191] | 2 | 46/M  51/F | FL  SLL | Single mass/head  Single mass/tail | Surgery+ chemotherapy（R-CHOP）  Surgery+ chemotherapy（R-CHOP） | ＞36 months/NED  ＞36 months/NED |
| Zafar Y et al [155] | 1 | 57/M | DLBCL | Single mass/tail | Chemotherapy | In remission |
| Yamai T et al [177] | 1 | 73/M | MCL | Multiple masses/  Head and tail | chemotherapy（R-CHOP） | 96 months/NED |
| Cagle BA et al [180] | 1 | 36/F | ALCL | Single mass/body | Chemotherapy (EPOCH) | NED |
| Rodríguez-Infante A et al [145] | 1 | 53/F | FL | Single mass/head | Surgery+ chemotherapy（R-CHOP）  + radiotherapy | 48 months/NED |
| [Pragalathan B](https://pubmed.ncbi.nlm.nih.gov/?term=B+P&cauthor_id=33401192) et al [146] | 1 | 71/M | BCL | Single mass/head | Surgical bypass + chemotherapy（R-CHOP） | NED |
| Ravi S et al [192] | 1 | 37/F | DLBCL | Single mass/head | chemotherapy（R-CHOP） | NA |
| Mimery A et al [196] | 1 | 63/F | DLBCL | Diffuse/ tail | chemotherapy（R-CHOP） | NA |
| Jones H et al [190] | 1 | 72/M | DLBCL | Single mass/tail | chemotherapy（R-CHOP） | Lost in follow-up |
| Fang YH et al [148] | 1 | 14/M | ALCL | Diffuse | chemotherapy | No remission |
| Badrinath M et al [203] | 1 | 69/M | BCL | Single mass/head | chemotherapy（R-CHOP） | In remission |
| Wu X et al [194] | 1 | 58/M | MALT  transformed into DLBCL | Multiple masses/  Neck and body | Surgery+ chemotherapy | Died 14 months after diagnosis/PD |
| Konjeti VR et al [187] | 1 | 68/F | BL | Single mass/head | chemotherapy | Die of spesis |
| León-Asuero-Moreno I et al [171] | 1 | 71/F | BCL | Single mass/body-tail | chemotherapy（R-CHOP） | NED |
| Baysal B et al [147] | 1 | 57/M | DLBCL | Single mass/head | chemotherapy（R-CHOP） | In remission |
| Zhu NX et al [176] | 5 | 57.4(49-67)  M/F 3/2 | BCL | Single/multiple 3/2  Head 5 | Chemotherapy 3  Chemotherapy+ radiotherapy 1  No treatment 1 | Survivors: 31.5 months/SD 2  Die of the disease 2  1 lost in follow-up |
| Liu W et al [181] | 1 | 62/M | TCL | Single mass/head | Surgery | Died 6 months after surgery |
| Wallace D et al [202] | 1 | 76/F | BCL | Single mass/head | Chemotherapy | In remission |
| Sood V et al [183] | 1 | 7/NA | DLBCL | Single mass/head | Chemotherapy | NA |
| Zheng SM et al [182] | 1 | 49/M | DLBCL | Single mass/head | Surgery+ chemotherapy（CHOP） | 60 months/NED |

* DLBCL: diffuse large B-cell lymphoma; HGBCL: high grade B-cell lymphoma; FL: follicular lymphoma; BL: Burkitt lymphoma; TCL: T cell lymphoma; MCL: Mantle Cell Lymphoma; ALCL: anaplastic large cell lymphoma; BCL: B-cell lymphoma; MALT: mucosa-associated lymphoid tissue lymphoma; SLL: small lymphocytic lymphoma; HL: Hodgkin lymphoma; NED: no evidence of disease; NA: no accessed; R-CHOP: rituximab, cyclophosphamide, doxorubicin, vincristine, and prednisone; CR: complete remission; PR: partial remission; SD: stable disease; PD: progressive disease; EPOCH: etoposide, prednisolone, oncovin, cyclophosphamide, and doxorubicin.
